# Trophic relations of *Opatrum sabulosum* (Coleoptera, Tenebrionidae) with leaves of cultivated and uncultivated species of herbaceous plants under laboratory conditions

**DOI:** 10.3897/zookeys.481.7015

**Published:** 2015-02-04

**Authors:** Viktor V. Brygadyrenko, Sergii S. Nazimov

**Affiliations:** 1 Department of Zoology and Ecology, Oles Honchar Dnipropetrovsk National University, Gagarin Avenue 72, Dnipropetrovsk 49010, Ukraine

**Keywords:** *Opatrum sabulosum*, Tenebrionidae, Food Preferences, Laboratory Experiments, Plant-eating Insects

## Abstract

We carried out a quantitative assessment of the consumption of herbaceous plants by *Opatrum sabulosum* (Linnaeus, 1761) – a highly significant agricultural pest species. We researched the feeding preferences of this pest species with respect to 33 uncultivated and 22 cultivated plant species. This species of darkling beetle feeds on many uncultivated plant species, including those with hairy leaves and bitter milky sap, such as *Scabiosa ucrainca* (5.21 mg/specimen/24 hours), *Euphorbia virgata* (3.45), *Solanum nigrum* (3.32), *Centauria scabiosa* (2.47), *Lamium album* (2.41), *Aristolochia clematitis* (1.76), *Chenopodium album* (1.73), *Arctium lappa* (1.51), *Asperula odorata* (1.20). A high rate of leaf consumption is also characteristic for cultivated species, for example, *Perilla nankinensis* (5.05 mg/specimen/24 hours), *Lycopersicon esculentum* (3.75), *Tropaeolum majus* (3.29), *Nicotiana tabacum* (2.66), *Rumex acetosa* (1.96), *Beta vulgaris* (1.27). *Opatrum sabulosum* is capable of feeding on plants which are poisonous to cattle. This species of darkling beetle consumes 95.5% of the cultivated and 48.5% of the uncultivated herbaceous plants researched.

## Introduction

For many species of phytophages and saprophages the consumption of leaves of herbaceous plants is the main aspect of their negative influence on natural ecosystems. If a particular species of insect feeds on one particular species of grass, it is quite easy to control its numbers in agricultural conditions (Fattorini 2011; Jia et al. 2013). The situation is much more difficult with polyphages potentially able to feed on many species of fodder plants (Whicker and Tracey 1987; Crawford 1988; Rogers et al. 1988; De Los Santos et al. 2002). *Opatrum sabulosum* (Linnaeus, 1761), a member of the Tenebrionidae family, is a pest species with a wide range of consumption preferences. This species has a wide distribution (Chernej 2005; Abdurahmanov and Nabozhenko 2011). It is numerous in the majority of steppe and meadow ecosystems, in pine forests and, most significantly, in agricultural ecosystems (Parmenter and Macmahon 1984; Minoranskij and Kuzina 1987). Its abilty to eat herbaceous plants from different families enables populations of this species to thrive in high and stable numbers over a long period of years in spite of all agro-technical measures directed against them (Kabanov and Sedin 1981; Leo et al. 2011).

The imagines of *Opatrum sabulosum* are most active in the first half of spring (Rejnhardt 1936). During this period it is usual to observe a few dozen to hundreds of this darkling beetle species in a single square meter plot. According to modern data the imagines of *Opatrum sabulosum* cause extensive damage to a large number of agricultural plant species, both on ploughed fields with wide furrows and densely sown fields with narrow gaps between the rows (Medvedev 1968). Newly planted pines suffer similar damage (Chernej 2005). According to Medvedev (1968) the imagines of *Opatrum sabulosum* prefer to feed on roots, the lower parts of stems and also root crops, making long, narrow passages in them. It is worth noting that these beetles readily eat the decaying parts of plants (Kabanov 1977; De Los Santos et al. 1988).

In natural conditions the imago of *Opatrum sabulosum* feeds on the leaves of steppe plants, and in fields it begins to damage both weeds and agricultural crops (Rejnhardt 1936). Cases of consumption of dry horse manure and dry remains of vegetation have been recorded (Chen et al. 2004). The peak of the feeding activity of *Opatrum sabulosum*, when it causes serious damage to sown crops, is observed at the end of April and in May (Dolin 1975).

The new generation emerges at the end of August (Knor 1975; Allsopp 1980; Carpaneto and Fattorini 2001). The number of actively feeding imagines declines considerably in mid summer (Naidu and Hattingh 1986; Gehrken and Somme 1994).

According to the information in the literature, the imagines of *Opatrum sabulosum* feed on wild and weedy species of plants in natural environments, and in agricultural ecosystems they transfer their consumption to cultivated plants and weeds. It is also widely assumed that this species of darkling beetle does not feed on species with bitter milky juice, such as *Euphorbia stepposa* Zoz ex Prokh. and *Cichorium intybus* L., and on hairy leaf species, such as *Agrimonia eupatoria* L. and *Asclepias syriaca* L., though statements on this point are fragmentary and require support.

Controlling the numbers of *Opatrum sabulosum* in its capacity as a highly significant pest on agricultural crops is impossible without a quantitative assessment of its consumption spectrum with respect to herbaceous plants. There is only fragmentary information in the scientific literature on the damage caused by this species to specific species of agricultural crops and this lacks a quantitative assessment of the amount of food consumed by an individual beetle. As a result of our preliminary research we have established that, though this species of darkling beetle has traditionally been considered a saprophage, its consumption of six types of soil and four types of steppe litter have not been observed in laboratory conditions (Nazimov and Brygadyrenko 2013). For this reason we consider *Opatrum sabulosum* to be a phytophage, feeding predominantly on herbaceous vegetation.

So, the following questions are of considerable interest: (1) whether *Opatrum sabulosum* eats the leaves of plants poisonous to cattle, (2) whether it eats green leaves of hairy plants, (3) whether the beetles prefer species from the natural flora or cultivated plants. In connection with these questions, the aim of this study is to establish in laboratory conditions the potential trophic relations of *Opatrum sabulosum* with the leaves of herbaceous plants belonging to different taxonomic groups.

## Materials and methods

The research was carried out on the outskirts of Dnipropetrovsk, Ukraine, at the end of July beginning of August 2013. A total of 1,920 *Opatrum sabulosum* individuals were collected on plots in steppe habitat and kept for 10 days on an optimal diet consisting of lettuce, cabbage and vine leaves. Sprinkling devices were placed in the containers so the beetles did not experience lack of water.

Each food item was offered in eight transparent plastic containers (8 × 12 × 10 cm) without any substrate provided, each with four beetles (two male, two female), a total of 32 imago specimens being involved in each experiment with a particular plant species. The temperature in the laboratory was maintained at 25–28 °C and the humidity at 60–80%. Each experiment lasted for five days. A control group was kept in 32 containers identical to those used in the main experiment, also without any substrate, but without any food, each holding four specimens.

The leaves of naturally occurring herbaceous plant species were collected from natural ecosystems which were not affected by anthropogenic pollution. The leaves of cultivated plants were collected from a private plot where the plants had been cultivated without the use of growth stimulators, herbicides, organic or mineral fertilisers. The green leaves were dried out over a period of 12 to 20 days in the open air on shelves in an open sided roofed structure. After this procedure the drying of the leaves was completed with a 24 hour period in a drying chamber.

To determine the mass of food consumed we took into account the degree of decomposition of the leaves under the influence of microorganisms. For this purpose, simultaneously with the main experiment we placed leaves of each plant species in eight identical containers (making a total of 440 containers) without *Opatrum sabulosum*. The consumption of food by the beetles was calculated using the optimised formula proposed by David (1998). All experiments were carried out in identical light, temperature and humidity conditions.

The weight of the food and the beetles was determined on analytical scales JD-100 (precision 1 mg). In the statistical analysis of the data we calculated *x* ± *S_x_*, the median and range of fluctuation for each characteristic. The most significant characteristic is the median (the normal distribution of the characteristics was not observed as the beetles do not eat daily in equal portions, but at an uneven rate, each “meal” varying considerably in the weight of food consumed).

## Results

### Consumption of leaves of wild herbaceous plant species

From the wild growing herbaceous plants imagines of *Opatrum sabulosum* consume predominantly the following species (Table 1): *Scabiosa ucranica* (5.21 mg/specimen/24 hours), *Euphorbia virgata* (3.45), *Solanum nigrum* (3.32), *Centaurea scabiosa* (2.47), *Lamium album* (2.41), *Aristolochia clematitis* (1.76), *Chenopodium album* (1.73), *Arctium lappa* (1.51), *Asperula odorata* (1.20). For the other plant species the intensity of food consumption did not exceed 1 mg per specimen over 24 hours. Among the above-mentioned species are both plants edible for cattle (*Chenopodium album* and *Centaurea scabiosa*) and species of plants which are not edible for the majority of phytophages (*Aristolochia clematitis*, *Euphorbia virgata* and *Solanum nigrum*).

**Table 1.** Consumption of leaves (mg/specimen/24 hours) of different species of uncultivated herbaceous plants by *Opatrum sabulosum* in laboratory conditions (*n* = 32).

Family	Species	Median	x ± S_x_	Min–Max
Apiaceae	*Aegopodium podagraria* L.	0.20	0.21 ± 0.11	0.04–0.35
Apocynaceae	*Vinca minor* L.	0.40	0.39 ± 0.27	0.04–0.77
Aristolochiaceae	*Aristolochia clematitis* L.	1.76	1.76 ± 0.75	0.65–3.00
Asclepiadaceae	*Asclepias syriaca* L.	0.18	0.18 ± 0.10	0.05–0.35
Asteraceae	*Arctium lappa* L.	1.51	1.42 ± 0.59	0.00–2.13
–“–	*Arctium tomentosum* Mill.	0.21	0.25 ± 0.20	0.00–0.71
–“–	*Artemisia absinthium* L.	0.40	0.36 ± 0.22	0.00–0.65
–“–	*Cirsium vulgare* (Savi) Ten.	0.88	0.79 ± 0.31	0.00–1.03
–“–	*Cichorium intybus* L.	0.60	0.64 ± 0.77	0.10–2.55
–“–	*Centaurea scabiosa* L.	2.47	2.47 ± 0.84	1.50–4.30
–“–	*Hieracium pilosella* L.	0.85	0.86 ± 0.58	0.15–2.15
–“–	*Senecio vernalis* Waldst. & Kit.	0.16	0.16 ± 0.09	0.00–0.25
Cannabaceae	*Humulus lupulus* L.	0.58	0.72 ± 0.56	0.00–1.63
Chenopodiaceae	*Chenopodium album* L.	1.73	2.03 ± 1.74	0.00–5.52
Convallariaceae	*Convallaria majalis* L.	0.54	0.44 ± 0.39	0.00–1.08
Dipsacaceae	*Scabiosa ucranica* L.	5.21	5.21 ± 1.31	3.10–7.45
Euphorbiaceae	*Euphorbia stepposa* Zoz ex Prokh.	0.74	0.74 ± 0.21	0.40–1.15
–“–	*Euphorbia virgata* W.K.	3.45	3.86 ± 1.76	2.50–8.30
Fabaceae	*Astragalus borysthenicus* Klokov.	0.55	0.55 ± 0.27	0.00–0.94
–“–	*Medicago romanica* Prodan.	0.95	1.11 ± 0.56	0.40–2.05
Hypericaceae	*Hypericum perforatum* L.	0.60	0.60 ± 0.30	0.00–1.15
Lamiaceae	*Ajuga genevensis* L.	0.25	0.62 ± 0.73	0.15–2.40
–“–	*Lamium album* L.	2.41	2.49 ± 1.36	0.00–5.31
–“–	*Salvia nemorosa* L.	0.60	0.58 ± 0.34	0.00–1.05
–“–	*Thymus marschallianus* Willd.	0.65	1.02 ± 1.25	0.15–4.25
Papaveraceae	*Chelidonium majus* L.	0.55	0.55 ± 0.30	0.16–1.04
Polygonaceae	*Polygonum aviculare* L.	0.70	0.87 ± 0.78	0.00–2.31
Rosaceae	*Agrimonia eupatoria* L.	0.08	0.08 ± 0.05	0.00–0.15
–“–	*Fragaria vesca* L.	0.15	0.17 ± 0.10	0.00–0.35
–“–	*Potentilla argentea* L.	0.35	0.35 ± 0.20	0.10–0.60
Rubiaceae	*Asperula odorata* L.	1.20	0.89 ± 0.65	0.00–1.60
Solanaceae	*Solanum nigrum* L.	3.32	3.15 ± 1.67	0.00–5.14
Violaceae	*Viola tricolor* L.	0.36	0.38 ± 0.31	0.00–0.97

In various containers the maximum speeds of food consumption significantly exceeded the average figures for each plant species, an effect most likely connected with the prolonged reproductive period of individual beetles and the intensive consumption of food for the development of eggs.

The following species were practically not consumed by *Opatrum sabulosum*: *Cirsium vulgare* (0.88), *Euphorbia stepposa* (0.74), *Hypercium perforatum* (0.60), *Salvia nemorosa* (0.60), *Astragalus borysthenicus* (0.55), *Chelidonium majus* (0.55), *Convallaria majalis* (0.54), *Artemisia absinthium* (0.40), *Vinca minor* (0.40), *Viola tricolor* (0.36), *Potentilla argentea* (0.35), *Arctium tomentosum* (0.21), *Aegopodium podagraria* (0.20), *Asclepias syriaca* (0.18), *Senecio vernalis* (0.16), *Fragaria vesca* (0.15) and *Agrimonia eupatoria* (0.08). For this group of plants the maximum rate of food consumption for any container did not exceed 1.2 mg per specimen per 24 hours.

The decrease in beetles’ body weight during the experiment (Table 2) is connected, first of all, with loss of moisture (during the entire five day period of the experiment the *Opatrum sabulosum* imagines did not have access to water). The control group of *Opatrum sabulosum*, denied access to food, decreased in body weight by 1.02 ± 0.27 mg/specimen/24 hours (2.05% over 24 hours). A larger decrease in body weight relative to the control group can be connected with the purgative effect on the intenstines of beetles from compounds contained in the food plants, intoxication of their organs or damage to their intestinal walls. This effect was observed for the following species; *Vinca minor* (–1.85 mg/24 hours), *Cichorium intybus* (–1.60), *Asperula odorata* (–1.44), *Solanum nigrum* (–1.44), *Salvia nemorosa* (–1.35), *Cirsium vulgare* (–1.20), *Potentilla argentea* (–1.20), *Euphorbia stepposa* (–1.19), *Euphorbia virgata* (–1.14), *Artemisia absinthium* (–1.14).

**Table 2.** Changes in body weight (mg/specimen/24 hours) of *Opatrum sabulosum* on diet of different uncultivated herbaceous plant species in laboratory conditions (*n* = 32).

Family	Species	Median	x ± S_x_	Min–Max
Apiaceae	*Aegopodium podagraria* L.	–0.33	–0.33 ± 0.31*	–1.00–+0.15
Apocynaceae	*Vinca minor* L.	–1.85	–2.41 ± 2.09*	–6.25––0.50
Aristolochiaceae	*Aristolochia clematitis* L.	–0.65	–0.69 ± 0.25*	–1.20––0.35
Asclepiadaceae	*Asclepias syriaca* L.	–0.95	–0.89 ± 0.12	–1.05––0.65
Asteraceae	*Arctium lappa* L.	–0.80	–0.73 ± 0.18*	–1.05––0.40
–“–	*Arctium tomentosum* Mill.	–0.67	–0.67 ± 0.19*	–0.95––0.40
–“–	*Artemisia absinthium* L.	–1.14	–1.14 ± 0.30	–1.45––0.55
–“–	*Cirsium vulgare* (Savi) Ten.	–1.20	–1.14 ± 0.14*	–1.35––0.85
–“–	*Cichorium intybus* L.	–1.60	–1.66 ± 1.19*	–4.15–+0.25
–“–	*Centaurea scabiosa* L.	–0.55	–0.60 ± 0.14*	–0.80––0.45
–“–	*Hieracium pilosella* L.	–0.95	–0.95 ± 0.24	–1.35––0.65
–“–	*Senecio vernalis* Waldst. & Kit.	–0.90	–0.94 ± 0.29	–1.60––0.65
Cannabaceae	*Humulus lupulus* L.	–1.05	–0.94 ± 0.29	–1.20––0.40
Chenopodiaceae	*Chenopodium album* L.	–0.65	–0.71 ± 0.27*	–1.25––0.40
Convallariaceae	*Convallaria majalis* L.	–0.80	–0.72 ± 0.36*	–1.20––0.15
Dipsacaceae	*Scabiosa ucranica* L.	–0.53	–0.53 ± 0.24*	–0.95––0.25
Euphorbiaceae	*Euphorbia stepposa* Zoz ex Prokh.	–1.19	–1.19 ± 0.40*	–2.00––0.75
–“–	*Euphorbia virgata* W.K.	–1.14	–1.14 ± 0.40	–1.75––0.65
Fabaceae	*Astragalus borysthenicus* Klokov.	–0.25	–0.21 ± 0.25*	–0.55–+0.25
–“–	*Medicago romanica* Prodan.	–0.80	–0.77 ± 0.19*	–0.95––0.40
Hypericaceae	*Hypericum perforatum* L.	–0.65	–0.48 ± 0.55*	–0.80–+0.95
Lamiaceae	*Ajuga genevensis* L.	–0.95	–0.88 ± 0.44	–1.45–+0.15
–“–	*Lamium album* L.	–0.79	–0.79 ± 0.19*	–1.20––0.55
–“–	*Salvia nemorosa* L.	–1.35	–1.47 ± 0.47*	–2.15––0.95
–“–	*Thymus marschallianus* Willd.	–1.05	–1.05 ± 0.42	–1.70––0.40
Papaveraceae	*Chelidonium majus* L.	–0.90	–1.06 ± 0.49	–2.20––0.55
Polygonaceae	*Polygonum aviculare* L.	–0.86	–0.86 ± 0.27*	–1.20––0.40
Rosaceae	*Agrimonia eupatoria* L.	–1.06	–1.06 ± 0.35	–1.55––0.55
–“–	*Fragaria vesca* L.	–0.68	–0.68 ± 0.16*	–0.95––0.40
–“–	*Potentilla argentea* L.	–1.20	–1.14 ± 0.34	–1.60––0.55
Rubiaceae	*Asperula odorata* L.	–1.44	–1.44 ± 0.41*	–2.15––0.75
Solanaceae	*Solanum nigrum* L.	–1.44	–1.44 ± 0.41*	–2.15––0.75
Violaceae	*Viola tricolor* L.	–0.66	–0.66 ± 0.22*	–1.05––0.40

Note: *–loss of body weight, observed at the end of the experiment, considered reliable at *P* < 0.05 differs from the body weight of the control group of beetles without access to food (1.02 ± 0.27 mg/specimen/24 hours); average body weight of beetles at start of experiment –49.61 ± 7.86 mg).

The minimum loss in body weight of *Opatrum sabulosum* compared to the start of the experiment was observed for the following species: *Astragalus borysthenicus* (–0.25 mg/specimen/24 hours), *Aegopodium podagraria* (–0.33), *Scabiosa ucrainca* (–0.53), *Centaurea scabiosa* (–0.55), *Aristolochia clematitis* (–0.65), *Chenopodium album* (–0.65), *Hypericum perforatum* (–0.65), *Viola tricolor* (–0.66), *Arctium tomentosum* (–0.67), *Fragaria vesca* (–0.68), *Lamium album* (–0.79), *Medicago romanica* (–0.80), *Convallaria majalis* (–0.80) and *Arctium lappa* (–0.80).

Maximum faecal formation by the beetles was observed following diets of *Polygonum aviculare* (0.73) and *Solanum nigrum* (0.70), and the minimum rate (equal to 0 mg/specimen/24 hours in all eight experimental containers) was observed after feeding on *Convallaria majalis* and *Vinca minor*. The intensity of faecal formation was at an intermediate level with the other plant species tested.

It is interesting that for *Aegopodium podagraria* one of the minimum rates of food consumption was observed (0.20 mg/specimen/24 hours), one of the minimum losses of body weight compared to the control group of beetles (–0.33 compared to –1.02 mg/specimen/24 hours for the group without access to food) and also the minimum rates of excrement formation (0.10 mg/specimen/24 hours). Thus, from 0.20 mg of food consumed per day 0.10 mg of excrement was formed, the remainder being expended on anabolism and respiration.

### Consumption of leaves of cultivated herbaceous plant species

The leaves of cultivated herbaceous plant species were consumed on average with the same intensity as the leaves of wild plant species (Table 3). The rates of leaf consumption were highest for the following species: *Perilla nankinensis* (5.05 mg/specimen/24 hours), *Lycopersicon esculentum* (3.75), *Tropaeolum majus* (3.29), *Nicotiana tabacum* (2.66), *Rumex acetosa* (1.96), *Beta vulgaris* (1.27).

**Table 3.** Consumption of leaves (mg/specimen/24 hours) of different cultivated herbaceous plant species by *Opatrum sabulosum* in laboratory conditions (*n* = 32).

Family	Species	Median	x ± S_x_	Min–Max
Apiaceae	*Daucus carota* L.	0.80	0.80 ± 0.47	0.00–1.74
Asteraceae	*Echinacea purpurea* (L.) Moench.	0.29	0.49 ± 0.61	0.00–2.04
–“–	*Matricaria recutita* L.	0.54	0.65 ± 0.50	0.00–1.54
–“–	*Helianthus annuus* L.	1.05	1.05 ± 0.26	0.77–1.55
–“–	*Helianthus tuberosus* L.	0.32	0.36 ± 0.26	0.00–0.77
Boraginaceae	*Borago officinalis* L.	0.86	1.05 ± 1.13	0.00–2.97
Chenopodiaceae	*Beta vulgaris* L.	1.27	1.49 ± 0.73	0.58–2.82
Cucurbitaceae	*Citrullus lanatus* (Thunb.) Matsum. & Nakai.	0.61	0.76 ± 0.68	0.00–1.89
–“–	*Cucurbita pepo* L.	0.37	0.37 ± 0.25	0.00–0.76
Lamiaceae	*Perilla nankinensis* (Lour.) Decne.	5.05	4.60 ± 1.75	0.00–5.66
Malvaceae	*Malva erecta* J. Presl & C. Presl	1.13	1.13 ± 0.51	0.32–1.74
Onagraceae	*Oenothera biennis* L.	0.73	0.96 ± 0.77	0.23–2.82
Phytolaccaceae	*Phytolacca americana* L.	0.49	0.57 ± 0.47	0.03–1.23
Poaceae	*Zea mays* L.	0.17	0.17 ± 0.08	0.02–0.31
Polemoniaceae	*Phlox paniculata* L.	0.44	0.44 ± 0.13	0.26–0.56
Polygonaceae	*Rumex acetosa* L.	1.96	2.00 ± 1.52	0.00–4.36
Ranunculaceae	*Aquilegia vulgaris* L.	0.68	0.63 ± 0.38	0.05–1.03
Rosaceae	*Fragaria moschata* (Duchesne) Weston.	0.24	0.87 ± 1.71	0.00–5.38
Solanaceae	*Capsicum annuum* L.	1.10	1.25 ± 0.40	0.82–2.04
–“–	*Lycopersicon esculentum* Mill.	3.75	3.74 ± 2.66	0.00–8.20
–“–	*Nicotiana tabacum* L.	2.66	3.05 ± 2.72	0.00–9.08
Tropaeolaceae	*Tropaeolum majus* L.	3.29	3.10 ± 2.23	0.00–6.54

Leaves of the following species were those consumed least intensively by *Opatrum sabulosum*: *Oenothera biennis* (0.73), *Aquilegia vulgaris* (0.68), *Citrullus lanatus* (0.61), *Matricaria recutita* (0.54), *Phytolacca americana* (0.49), *Phlox paniculata* (0.44), *Cucurbita pepo* (0.37), *Helianthus tuberosus* (0.32), *Echinacea purpurea* (0.29), *Fragaria moschata* (0.24) and *Zea mays* (0.17 mg/specimen/24 hours). It is interesting that of all the cultivated grasses researched, the minimum quantity of dried leaves was consumed for maize despite the fact that this is the main crop damaged by *Opatrum sabulosum*. It is worth emphasising once again that phytophages eat the fresh or decaying leaves of this species but hardly ever dry leaves.

Compared to the control group without access to food, for which we observed a decrease in body weight of 1.02 ± 0.27 mg/specimen/24 hours, the consumption of many species of cultivated plants minimises the loss of the original body weight. This can be seen with *Daucus carota* (–80 mg/specimen/24 hours), *Nicotiana tabacum* (–0.80), *Phlox paniculata* (–0.80), *Capsicum annuum* (–0.76), *Phytolacca americana* (–0.75), *Helianthus tuberosus* (–0.74), *Malva erecta* (–0.74), *Oenothera biennis* (–0.70), *Rumex acetosa* (–0.67), *Lycopersicon esculentum* (–0.65), *Fragaria moschata* (–0.64), *Helianthus annuus* (–0.58), *Matricaria recutita* (–0.55), *Zea mays* (–0.55), *Citrullus lanatus* (–0.53), *Aquilegia vulgaris* (–0.49), *Tropaeolum majus* (–0.40), *Cucurbita pepo* (–0.25) and *Borago officinalis* (–0.20). In our experiment the consumption of dry leaves of *Beta vulgaris* did not lead to a reliable tendency towards preservation in the beetles’ body weight compared to the control group (–1.08 and –1.02 mg/specimen/24 hours respectively).

The maximum intensity of faecal formation for *Opatrum sabulosum* was characteristic for diets of dry leaves of the following species: *Daucus carota* (1.03 mg/specimen/24 hours), *Lycopersicon esculentum* (0.65), *Fragaria moschata* (0.55), *Perilla nankinensis* (0.55), *Citrullus lanatus* (0.53), *Rumex acetosa* (0.45), *Nicotiana tabacum* (0.41), *Zea mays* (0.41), *Capsicum annuum* (0.30), *Cucurbita pepo* (0.30) and *Helianthus annuus* (0.30).

## Discussion

The research showed that with 16 of the 33 wild and 21 of the 22 cultivated herbaceous plant species investigated the species of leaf consumed led to a reliable loss in the beetles’ body weight (see Tables 2 and 4). Overall, *Opatrum sabulosum* consumed 95.5% of the cultivated and 48.5% of the wild herbaceous plant species researched. Of the 11 species in the Asteraceae family 8 were consumed, of the 5 Lamiaceae species 4 were consumed, of the 4 Rosaceae species 1 was consumed and of the 4 Solanaceae species 1 was consumed. Representatives of the following families included in our research were not consumed at all: Apocynaceae, Asclepiadaceae, Convallariaceae, Hypericaceae, Papaveraceae, Rosaceae and Violaceae.

**Table 4.** Changes in body weight (mg/specimen/24 hours) of *Opatrum sabulosum* on diet of leaves of different cultivated herbaceous plant species in laboratory conditions (*n* = 32).

Family	Species	Median	x ± S_x_	Min–Max
Apiaceae	*Daucus carota* L.	–0.80	–0.87 ± 0.40*	–1.60––0.40
Asteraceae	*Echinacea purpurea* (L.) Moench.	–0.95	–0.95 ± 0.39	–1.85––0.40
–“–	*Matricaria recutita* L.	–0.55	–0.66 ± 0.23*	–1.20––0.40
–“–	*Helianthus annuus* L.	–0.58	–0.58 ± 0.21*	–0.80––0.15
–“–	*Helianthus tuberosus* L.	–0.74	–0.74 ± 0.36*	–1.35––0.25
Boraginaceae	*Borago officinalis* L.	–0.20	–0.22 ± 0.28*	–0.80–+0.15
Chenopodiaceae	*Beta vulgaris* L.	–1.08	–1.08 ± 0.30	–1.65––0.55
Cucurbitaceae	*Citrullus lanatus* (Thunb.) Matsum. & Nakai.	–0.53	–0.53 ± 0.15*	–0.80––0.40
–“–	*Cucurbita pepo* L.	–0.25	–0.34 ± 0.19*	–0.65––0.15
Lamiaceae	*Perilla nankinensis* (Lour.) Decne.	–0.95	–0.96 ± 0.32	–1.45––0.55
Malvaceae	*Malva erecta* J. Presl & C. Presl.	–0.74	–0.74 ± 0.20*	–1.05––0.40
Onagraceae	*Oenothera biennis* L.	–0.70	–0.70 ± 0.40*	–1.45––0.15
Phytolaccaceae	*Phytolacca americana* L	–0.75	–0.74 ± 0.20*	–1.05––0.40
Poaceae	*Zea mays* L.	–0.55	–0.54 ± 0.18*	–0.80––0.20
Polemoniaceae	*Phlox paniculata* L.	–0.80	–0.96 ± 0.43*	–2.00––0.40
Polygonaceae	*Rumex acetosa* L.	–0.67	–0.67 ± 0.27*	–1.05––0.20
Ranunculaceae	*Aquilegia vulgaris* L.	–0.49	–0.49 ± 0.46*	–1.05–+0.55
Rosaceae	*Fragaria moschata* (Duchesne) Weston.	–0.64	–0.64 ± 0.33*	–1.35––0.25
Solanaceae	*Capsicum annuum* L.	–0.76	–0.76 ± 0.22*	–1.20––0.55
–“–	*Lycopersicon esculentum* Mill.	–0.65	–0.65 ± 0.14*	–0.80––0.40
–“–	*Nicotiana tabacum* L.	–0.80	–0.72 ± 0.16*	–0.95––0.40
Tropaeolaceae	*Tropaeolum majus* L.	–0.40	–0.43 ± 0.31*	–0.80–+0.25

Note: *–see Table 2.

According to the work of Rejnhardt (1936) *Opatrum sabulosum* eats the roots and leaves of wild steppe weeds such as *Atriplex hortensis* L., *Chenopodium album* L., *Convolvulus arvensis* L. and *Polygonum aviculare* L. Our experiments have confirmed that imagines of *Opatrum sabulosum* do feed on weed species. However, they do not show a clear preference for this type of food compared to cultivated plants. Indeed, the lowest decreases in original body weight were observed when the beetles fed on cultivated plants such as *Zea mays*, *Daucus carota*, *Cucurbita pepo*, *Helianthus annuus*, *Helianthus tuberosus*, *Borago officinalis* and *Nicotiana tabacum*. At the same time consumption of weeds also helps to reduce the loss of original body weight. Based on the results of our research, we can state that *Opatrum sabulosum* damages in almost equal ratios both cultivated plants and weeds.

*Opatrum sabulosum* has shown an ability to feed on species of plants with hairy leaves and a bitter milky sap. The beetles lost hardly any weight when feeding on the bitter leaves of *Astragalus borysthenicus* and *Aristolochia clematitis*, and also experienced insignificant weight loss in variants of the experiments with hairy plant species such as *Scabiosa ucrainca* and *Centauria scabiosa*. It follows that this species of darkling beetle consumes a fairly wide range of bitter species not eaten by livestock.

According to modern data (Kabanov 1977; Kabanov and Sedin 1981), the imagines of *Opatrum sabulosum* damage quite a large number of agricultural plants, including *Hordeum sativum* L., *Avena sativa* L., *Panicum virgatum* L., *Triticum durum* L., *Cicer arietnum* L., *Lens culinaris* Medikus., *Phaseolus vulgaris* L., *Sorghum saccharatum* (L.) Moench, *Sorghum bicolor* (L.) Moench, *Zea mays*, *Allium cepa* L., *Nicotiana tabacum*, *Solanum tuberosum* L., *Lycopersicon esculentum*, *Beta vulgaris*, *Helianthus annuus*, *Cannabis sativa* L., *Perilla nankinensis*, *Brassica napus* L., *Papaver somniferum* L., *Citrullus lanatus* and *Cucumis sativus* L. (Bryzova and Kelejnikova 1964; Medvedev 1968). They also damage the leaves of *Vitis vinifera* L., and eat the cotyledons of shoots of fruit trees.

It is clear that outside the reproductive period *Opatrum sabulosum* is able to feed intensively on both wild and cultivated species of herbaceous plants. According to information from the literature (Parmenter et al. 1989a, 1989b; Semida et al. 2001), there is considerable seasonal change in the diet of this species of darkling beetle (Cloudsley-Thompson 1975): this is connected both with the passage of definite phenological phases in the development of herbaceous plants (shoots, the formation of leaf rosettes near the roots), and with the spring reproductive period of the beetles themselves. Nevertheless, during the period of decreased trophic activity in the second half of summer (our experiment was carried out in late July – early August) the trophic activity of *Opatrum sabulosum* imagines continued at a pretty high level.

The seasonal dynamic of the trophic activity of this species of darkling beetle requires further research, especially the characteristics of its trophic activity (the quantitative and qualitative differences in its diet) during the period of intensive spring feeding and during the egg laying period. The peculiarities of the larval consumption of the root parts of wild and cultivated plants requires detailed research. The sex and age differences in the diet of the beetles in their first and second years of life remain unstudied. Besides this, the differences in the consumption of dry, fresh and decaying leaves of the beetle’s main species of food plants are of considerable interest. The results of studies of the chemical content of the plants consumed by *Opatrum sabulosum* will form the basis for the construction of models of the trophic relations of this species of polyphage, which is one of the most intensively studied and economically significant species of insect.
